# The Influence of Fiber-Form Waste Tire Aggregates on the Flexural Strength, Ductility, and Energy Dissipation of Pultruded GFRP–Rubberized Concrete Hybrid Beams

**DOI:** 10.3390/polym17243274

**Published:** 2025-12-10

**Authors:** Ali Serdar Ecemis, Memduh Karalar, Alexey N. Beskopylny, Sergey A. Stel’makh, Evgenii M. Shcherban’, Ceyhun Aksoylu, Emrah Madenci, Yasin Onuralp Özkılıç

**Affiliations:** 1 Department of Civil Engineering, Necmettin Erbakan University, 42090 Konya, Türkiye; asecemis@erbakan.edu.tr; 2 Department of Civil Engineering, Faculty of Engineering, Zonguldak Bulent Ecevit University, 67100 Zonguldak, Türkiye; memduhkaralar@beun.edu.tr; 3 Department of Transport Systems, Faculty of Roads and Transport Systems, Don State Technical University, 344003 Rostov-on-Don, Russia; besk-an@yandex.ru; 4 Department of Unique Buildings and Constructions Engineering, Don State Technical University, 344003 Rostov-on-Don, Russia; sergej.stelmax@mail.ru; 5 Department of Engineering Geometry and Computer Graphics, Don State Technical University, 344003 Rostov-on-Don, Russia; au-geen@mail.ru; 6 Department of Civil Engineering, Konya Technical University, 42250 Konya, Türkiye; caksoylu@ktun.edu.tr; 7 Department of Technical Sciences, Western Caspian University, Baku 1001, Azerbaijan

**Keywords:** fiber waste rubber, concrete core pultruded profile, hybrid beam, bending strength, composite

## Abstract

This study investigates the effects of different proportions of waste rubber fiber aggregates on the flexural behavior of reinforced concrete beams. Beam specimens were prepared with different proportions (5%, 10%, and 15%) of waste rubber fiber aggregates, and composite beams formed with pultruded GFRP profiles were tested under vertical load. According to the results of this study, cube compressive strength, cylinder tensile strength, and beam flexural strength decreased by 27.5%, 50%, and 47.6%, respectively, with the use of a 15% waste rubber aggregate. As a result of the four-point bending tests performed on reinforced concrete beams, the maximum load-carrying capacity of the beams decreased significantly after increasing the waste rubber aggregate ratio to 10% and 15%. However, a general improvement in the ductility of the beams was observed. One of the main results of this study is that when the waste rubber aggregate content is 5%, the best balance between strength and ductility is achieved, and the performance closest to the reference beams is obtained. The tests also revealed that the Ø10-5% specimen exhibited higher performance in terms of both load-carrying capacity and yield stiffness. On the other hand, although the 15% waste rubber aggregate ratio caused a decrease in the maximum load-carrying capacity. along with an increase in the diameter of the tensile reinforcement, this decrease was quite low. Finally, an overall decrease in energy consumption capacity was observed with increasing waste rubber aggregate content in all test beams. This can be attributed to the acceleration of shear damage in the beam and the shrinkage of the area under the load–displacement curve as the amount of waste increases. Additionally, SEM analyses were conducted in order to investigate the microstructural behavior of the rubberized concrete. This study has shown that the use of waste rubber aggregates can be environmentally and economically beneficial, especially at the 5% level.

## 1. Introduction

The most popular building material that uses a lot of natural resources, concrete, has been the subject of research on the use of waste tires in concrete manufacturing processes in recent years. Researchers from all over the world have been interested in rubberized concrete, which is created by adding crushed scrap tires to regular concrete. As a result, research has moved to areas such as structural interactions, fatigue performance, impact performance, freeze/thaw performance, and durability performance. Research has shown that structural components made of concrete with low levels of rubber may be used often [1]. Several studies have been undertaken to assess the impact of rubber on concrete. Recycled tire rubber has been suggested as a potential substitute for cement and natural aggregates in conventional concrete mixes to address environmental concerns about tire destruction. Hence, using scrap tire rubber in concrete may provide sustainability benefits while ensuring economic feasibility. Using recycled tire rubber as a partial replacement for fine aggregates in cement concrete is an attempt to mitigate environmental damage and preserve natural resources [2]. A significant amount of research is dedicated to investigating the use of tire rubber as an aggregate in order to manufacture environmentally friendly concrete [3]. The use of used tires to make concrete is becoming more commonplace. Rubber may be used to replace aggregates in regular concrete, roller-compacted concrete, self-compacting concrete, and high-strength concrete [4]. The size, proportions, and surface roughness of the rubber particles have a significant impact on the compressive strength of rubberized concrete mixes, as shown in investigations by Eldin & Senouci [5] and Topcu [6]. Shahjalal et al. [7] examined the combined impacts of polypropylene (PP) fibers, recycled coarse aggregates, and crumb rubber on the mechanical and physical characteristics of fiber-reinforced rubberized recycled concrete. Moreover, their study examined the bending behavior of reinforced concrete beams. The experiment revealed that the use of 30% recycled coarse aggregates, 5% crumb rubber, and 0.5% polypropylene fibers in concrete beams resulted in enhanced flexural capacity, ductility, and toughness. Vadivel et al. [8] examined the use of scrap tire rubber as a strengthening material in inadequately reinforced beams. For this specific objective, a total of 14 beams were created using two pieces of waste tire rubber measuring 15 mm × 25 mm as a substitute for steel reinforcement in the stress zone. Compared to traditionally reinforced beams, all rubber-reinforced beams had a more malleable behavior. A study conducted by Chan et al. [9] examined the effects of fine aggregate replacement ratios ranging from 0 to 75% by volume in FRP-confined rubber concrete (FCRC) using a variety of FRP thicknesses. The test findings made it clearer how the two crucial parameters—the replacement ratio of fine aggregates and the thickness of the FRP—affect rubber concrete’s axial behavior, thereby validating the efficiency of FRP confinement. Another study was performed by Li et al. [10]. Li et al. [10] examined the properties of concrete cylinders enclosed in FRP tubes and altered with waste tire rubber. The research demonstrates that FRP-tube-presented rubberized concrete cylinders have enhanced confinement effectiveness, ductility, and elastic regions in comparison to FRP-tube-encased conventional concrete cylinders. The concrete made using waste tire fibers has a superior performance compared to the less expensive crumb-rubber-modified concrete. The study conducted by Elshazlyc et al. [11] provided an overview of rubberized concrete mixes and their specific characteristics, including durability, flexibility, noise, and permeability to water, as well as resistance to acid and sulfate. Furthermore, the authors examined a critique about the use of rubberized concrete in structural components and its impact on the final compressive strength and ductility [11]. Rubberized concrete may be strengthened to a higher ultimate strength rating while retaining the flexibility that comes from the addition of rubber if it is contained by steel tubes or fiber-reinforced plastic (FRP). The study performed by Youssf et al. [12] investigates the potential use of crumb rubber concrete for structural columns. The researchers evaluated the effectiveness of using fiber-reinforced polymer confinement to address the limitations of the material. Based on the findings, it can be concluded that using fiber-reinforced polymers to contain rubberized concrete preserves the benefits of enhanced ductility while successfully negating the drop in strength. Nematzadeh et al. [13] performed another investigation. In their investigation, a comprehensive study in this field was carried out for the first time to examine the impact of steel fibers and crumb rubber (CR) aggregates on the shear performance of high-strength concrete beams that are reinforced with glass FRP (GFRP) bars. It was noted that increasing the ratio of fibers in terms of volume in the rubberized concrete beams with GFRP rebars resulted in a reduction in both the maximum width and the amount of fractures. Adday and Ali [14] demonstrated that the flexibility that disappears throughout the building of rubberized reinforced concrete beams may be regained. Considering this, the assessment most relevant to the research objective will involve comparing the control reinforced concrete beam, which was not externally strengthened or rubberized, with the rubberized reinforced concrete beams that were enhanced using single and double layers of carbon fiber-reinforced polymer (CFRP) sheets. The findings demonstrate that the load at the first fracture of the rubberized reinforced concrete beam matches that of the un-rubberized beam when strengthened with a single layer of carbon fiber-reinforced polymer (CFRP) sheets. Furthermore, the load rises by 20% when the beam is reinforced with two layers of CFRP sheets. A comparative study comparing flanged and non-flanged hollow and concrete-filled tubes was conducted by Ferdous et al. [15]. To further forecast the basic behavior of the beams, finite element modeling was carried out. The findings indicated that the addition of a concrete filler had a minor positive impact on bending performance while greatly improving the shear characteristics of the beam. When compared to tubes filled with regular concrete, the deformation and shear behavior of the beam is slightly impacted by the addition of 25% crumb rubber to the concrete. Batayneh et al. [16] suggested that waste tires may be effectively included into concrete mixtures at various proportions, such as 20%, 40%, 60%, 80%, and 100%, offering significant potential for usage. The test findings indicated that although the use of crumb tires may lead to a decrease in compressive strength, it still satisfies the strength criteria for light-weight concrete. Thomas et al. [17] performed another investigation. In their study, in high-strength cement concrete, natural fine particles were partially substituted with waste tire rubber in the form of crumb rubber. Fine aggregates were substituted with crumb rubber in multiples of 2.5%, ranging from 0% to 20%. The test findings indicate that the high-strength rubberized concrete exhibits a high level of resistance to harsh situations. In another investigation, Thomas et al. [18] reported the findings of a research investigation comparing the macrocell deterioration, resistance to acid attacks, and depth of chloride penetration of rubberized concrete and the control mix concrete. Abaqus was used to conduct analytical research, and the outcomes were compared against compressive and flexural strengths measured in a lab. Regarding compressive strength and flexural tensile strength, the Abaqus analytical findings demonstrate the same pattern as the laboratory experimental studies. Optimized step-by-step design calculations that take into consideration bending, shear, local/global buckling, and material failure for FRP beams were provided by Qiao et al. [19]. The design equations were presented in terms of panel apparent moduli and strengths, beam stiffness coefficients, and beam geometry. Qiao et al. [19] derived beam strength from a combined empirical and analytical examination of eight typical beams. These eight components were tested to confirm the design specifications, and the testing data was used to validate the design equations for bending/shear deflections and stresses, local and global buckling critical loads, and ultimate bending/shear strengths. A comprehensive design approach that addresses the most crucial aspects of FRP beam design was introduced. Madenci et al. [20] utilized the pultrusion process to manufacture pultruded GFRP profiles of various shapes. When exposed to different loads, pultruded GFRP profiles undergo form changes. A study was conducted to analyze the deflection and stress behavior of a pultruded GFRP composite beam at multiple scales. The outcomes of numerical and experimental research are used to validate the suggested formulations. When the analytical results were compared to the finite element and experimental outcomes, it was found that there was excellent agreement because the variations among the analytical results and the numerical and experimental outcomes were less than 1%. Equations for predicting critical flexural load capacities were proposed by Estep et al. [21]. The suggested model allowed for precise prediction of the failure mechanism and the load to failure for span-to-depth (L/h) proportions of roughly less than 42. Enhancing the precision of the laminate characteristics was expected to lead to improved accuracy in forecasting the key flexural load capacity. Karalar et al. [22] examined the effects of waste tire rubber on the flexural behavior of reinforced concrete beams. To achieve this objective, the volumetric ratios of waste tire rubber were selected to vary from 0% to 7.5% across the concrete beams. The beams using 2.5% waste tire rubber showed the greatest performance out of all the beams. Moreover, according to the test results, it can be observed that while the rate of waste tire rubber in the RCBs increases, the maximum bending metric in the RCBs also increases. The other investigation was performed by Zeng et al. [23]. Within the scope of their investigation, the utilization of fiber-reinforced polymer grids as reinforcements for ultra-high-performance concrete plates has been suggested. For the three-point bending tests, a total of 26 plates (600 × 120 × 40 mm) were manufactured. FRP grid–ultra-high-performance concrete composite plates’ flexural behavior was examined in relation to the fiber type, fiber content, and fiber length. When creating composite plates, it was discovered that using 12 mm long polyethylene fibers with a 1% volume fraction was cost-effective. In addition to the literature, there are also various studies available elsewhere [24,25].

### Aim of This Study

The previously mentioned studies have examined the impact of using scrap tire rubber as a waste material in concrete. Furthermore, the use of pultrusion composite profiles made of reinforced polymers filled with waste rubber concrete has also been investigated. However, the determination of the bending properties of this special, structurally integrated hybrid beam, which is created by incorporating a fiber-shaped waste rubber-reinforced concrete core and traditional steel reinforcement into a restrictive pultrusion GFRP profile, is quite limited.

The limited number of experiments carried out on waste rubber concrete-filled reinforced polymer (FRP) pultrusion hybrid shapes has been highlighted by this literature review. However, no studies on the bending properties of hybrid beams have been found. These beams were created and engineered by utilizing an inventive method that involves the use of waste rubber concrete-filled reinforced polymer (FRP) pultrusion composite profiles. This unique detail serves as a primary structural element to investigate complex mechanical interactions under bending loads, thereby methodologically differentiating itself from existing FRP–concrete hybrids. An important part of the issue is examined in this paper, which makes it noteworthy: the behavior of concrete-reinforcing parts under bending.

In recent times, there has been a significant surge in environmental consciousness, leading to a more frequent emphasis on the notion of eco-friendly green concrete. Thus, recycling tires is crucial for increasing awareness about the environment. Multiple studies and research efforts are conducted to enhance the effectiveness of reinforced concrete beams subjected to bending and to facilitate their restoration in the event of damage by using fiber reinforced polymer (FRP) reinforcement. Insufficient research has been conducted in the existing body of literature about the behavior of ecologically friendly green concrete core hybrid beams that include new-generation materials and waste materials.

The purpose of this study is to provide designers with an alternative perspective by establishing the bending strength standards for hybrid beams made from waste tire fibrous concrete core pultrusion profiles. This research is methodologically novel in terms of the following coupled performance analysis:

- Decreased compressive strength due to rubber addition;
- Increased ductility due to rubber addition;
- The constraint effect arising from the GFRP profile are simultaneously investigated under a bending load.

To achieve this objective, the concrete that was created from waste tires in the form of fibers at concentrations of 5%, 10%, and 15% was inserted into the pultrusion profile. A total of 12 trials were conducted. Even though the standards include the use of FRP composite materials, this study is still needed to determine how using this material with waste materials affects their strength and behavior. The purpose of this study is to close this gap in the literature.

## 2. Materials and Methods

This project aims to manufacture concrete by substituting natural aggregates with waste rubber in the form of rubber-based fibers with specific ratios of 5%, 10%, and 15%. After the aforementioned concrete is filled into box-shaped Pultruded GFRP profiles, bending experiments were conducted on beam specimens. Necmettin Erbakan University’s Department of Construction served as the location for the laboratory where the trials were conducted.

### Materials

**Figure 1:**
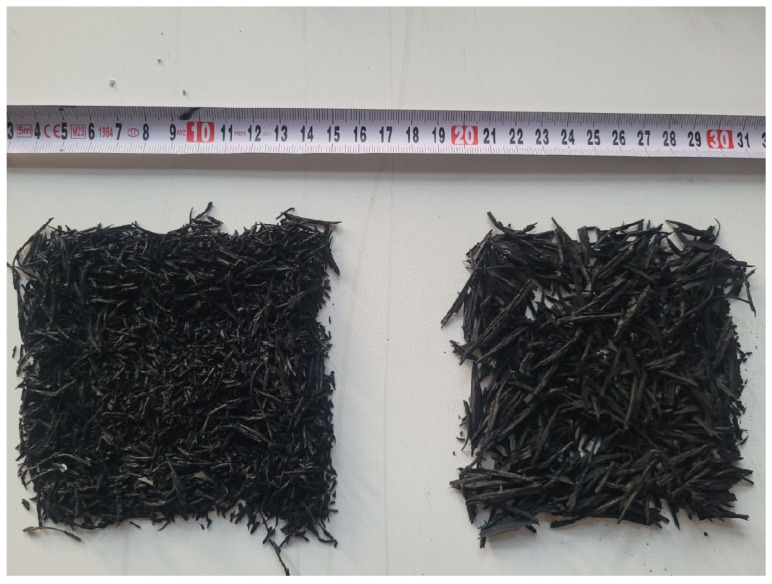
Components in the mixture.

The Konya area was the source of both the crushed stone coarse-grained normal-weight aggregate and the crushed stone fine-grained normal-weight aggregate that were evaluated in this research. Two sizes of the coarse-grained normal weight aggregate were used, namely 4–11.2 mm and 11.2–22.4 mm. Additionally, the fine-grained normal weight aggregate with a size of 0.4 mm was utilized. In the investigation, two types of rubber aggregates were utilized: coarse fibers, which went through a 16 mm filter and were left on a 4 mm screen, and fine fibers, which went through a 4 mm sieve. Figure 1 displays images of the rubber aggregates.

**Figure 2:**
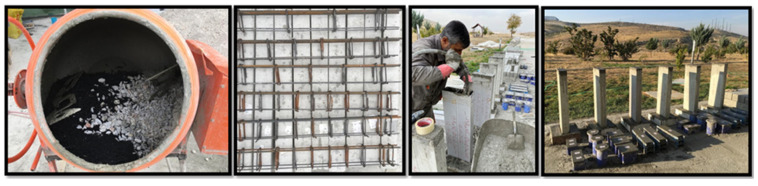
Stages involved in preparing the test samples.

**Figure 3:**
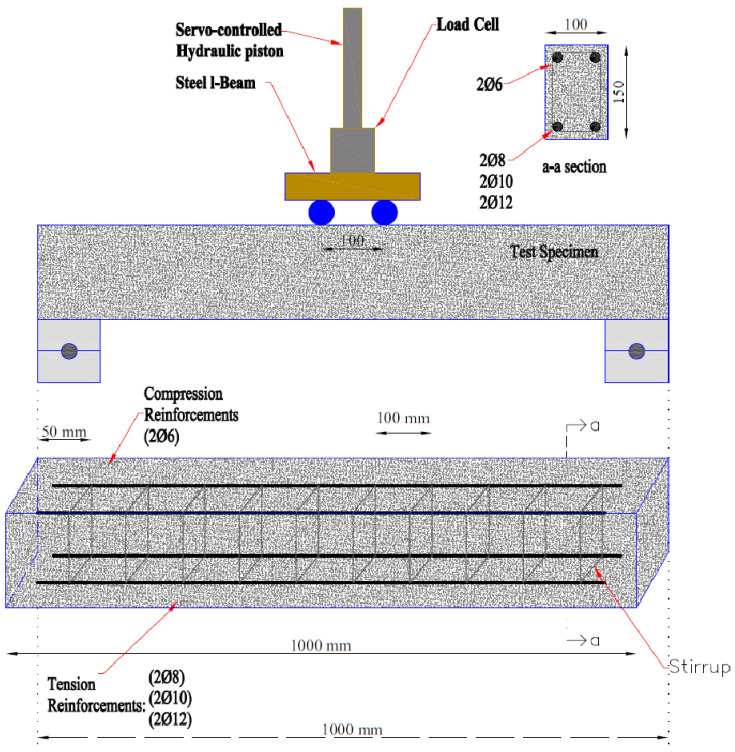
Test specimen and reinforcement layout.

Konya Cement Factory’s CEM I 42.5 R cement, with a specific surface of 3862 cm^2^⁄gr, was the product used in this investigation. The concrete additive utilized was Polisan 612, which is a hyperplasticizer based on polycarboxylate. CEM I 42.5 R cement’s chemical and physical characteristics are detailed in Table 1. This admixture enhances the strength of concrete by lowering the amount of water needed in the mixing process. Additionally, it greatly improves the workability time of new concrete. The water utilized at the structural testing laboratory of Necmettin Erbakan University, located in the Meram region of Konya area, was sourced from the main water supply. A concrete reference mixture was developed using Portland cement, aggregates, water, and plasticizers. Subsequently, rubberized concrete was manufactured by substituting the original particles in the mixture with discarded fiber-form waste tires in certain ratios. The tire replacement rates using aggregates amount to 5%, 10%, and 15% based on volume. The prepared combinations were placed into glass fiber-reinforced polymer (GFRP) profiles and left to cure for 28 days, which is the necessary period for the concrete to achieve its optimal strength. The proposed measurements for the bending beams to be manufactured are 100 × 150 × 1000 mm. Three distinct diameters (Ø8, Ø10, and Ø12) of longitudinal reinforcement were organized for the purpose of conducting bending tests. Stirrup spacing was selected as 100 mm in all specimens. For all beams to exhibit ductile behavior, the beam reinforcement ratio (ρ = 0.00125) was chosen to be lower than the balanced reinforcement percentage. It was determined that the stirrup volume ratio (ρ_w_) was 0.0057. The suitable parameters were determined to be a shear span with an effective depth of 2.69. Furthermore, this study considered three distinct fiber ratios: 5%, 10%, and 15%. The stages involved in preparing the test samples are given in Figure 2. As shown in Figure 3, all of the samples were examined under a four-point bending load. For each test, a beam sample was prepared and tested. Table 2 lists the average of the generated concrete’s cube, cylinder splitting, and beam bending strengths. Table 2 demonstrates that all concrete strengths declined as the percentage of used waste tire materials increased. However, as the rubber ratio employed increases, the actual weight of concrete per unit volume decreases. Due to its smaller unit volume weight, tires are utilized to replace the aggregates. All concrete strengths reduced with an increase in the percentage of fiber-formed waste tires used in concrete mixtures. In addition, as the proportion of waste rubber aggregates in the concrete increased, a decrease in the slump value of the concrete was observed. While the reference sample’s 28-day average cube compressive strength was 22.86 MPa, it was 12.65 MPa with 5% waste tire, 8.42 MPa with 10% waste tire, and 6.32 MPa with 15% waste tire. Strength decreased to 27.5% of the reference combination with 15% rubber. In terms of bending strength, the situations are comparable. Bending experiments determined that the reference beam’s bending strength was 8.27 MPa. The bending strength was 5.26 MPa for 5% waste rubber, 6.01 MPa for 10% waste rubber, and 4.33 MPa for 15% waste rubber. The results on tensile strength decreased with the increase in the proportion of used tires. The reference sample had a tensile strength of 2.31 MPa, while the tire ratios of 5%, 10%, and 15% had strengths of 1.58, 1.24, and 1.18 MPa, respectively. Strength dropped by 50% when the waste tire rate at 15%.

## 3. Results

### 3.1. Microstructure Analysis (SEM)

**Figure 4:**
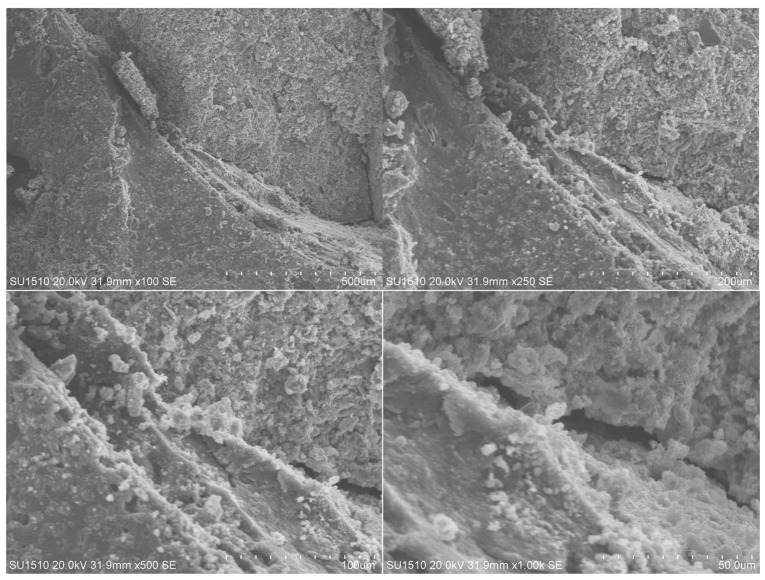
SEM analysis result.

**Figure 5:**
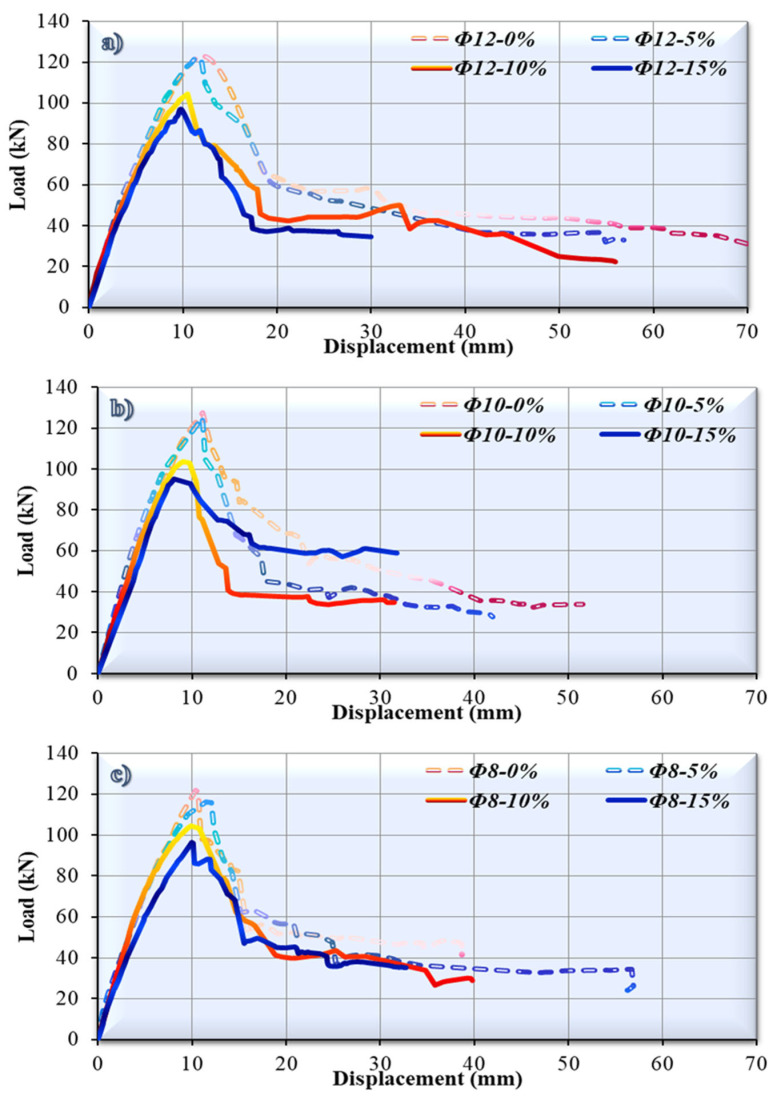
Graphs demonstrating the relationship between load and displacement for the tested specimens.

In this study, a concrete sample containing 5% waste rubber aggregates in their fibrous form was examined using a Scanning Electron Microscope (SEM) to determine its microstructural properties (Figure 4). Based on experimental data, this sample was determined to be the mixture that provided the best balance between flexural strength and ductility. According to SEM images, the microstructure is dense and compact, with rubber particles evenly distributed and homogeneously embedded within the cement paste. No visible voids or separations were observed in the transition zones (ITZs), indicating adequate rubber–cement interaction. Additionally, microcracks were observed to be rare in the sample, and the bond between the rubber fibers and the matrix was continuous. These structural properties support ductile behavior under loading and retard crack propagation. This is consistent with the total energy consumption and plastic deformation capacity shown in the load–displacement curves (Figure 5).

### 3.2. Effects of Varying Proportions of Waste Rubber Aggregates

As a consequence of the experiment, vertical load–displacement graphs were produced, and the damage occurring to the generated beam specimens was analyzed in order to investigate bending performance. Figure 5 presents a graphical representation of the results. By examining the load–displacement diagrams generated following the investigation, it was possible to determine that, in the experiments where the tensile reinforcement was chosen as Ø12, the maximum load level was attained at 123 kN at 12 mm displacement when there was no waste tire (reference beam-Ø12-0%). The greatest load carried and the amount of displacement related to this load were discovered to decline as the tire proportion rose, as observed in Figure 5a.

In the case of 5% (Ø12-5%), 0% (Ø12-10%), and 15% (Ø12-15%) tire proportions, the highest load that occurred and the values of displacement that corresponded to this load were found to be 11.6 mm and 122.12 kN (Ø12-5%), 10.5 mm and 104.24 kN (Ø12-10%), and 9.87 mm and 96.72 kN (Ø12-15%), respectively. In addition, when the stiffness values corresponding to the maximum value were examined, it was found that as the waste rubber aggregate ratio increased, stiffness decreased except for the Ø12-5% specimen. The stiffness of each specimen corresponding to the maximum load value was obtained as 10.25 kN/mm (Ø12-0%), 10.52 kN/mm (Ø12-5%), 9.92 kN/mm (Ø12-10%), and 9.80 kN/mm (Ø12-15%). When these stiffness values are compared with the Ø12-0% reference specimen, an increase of 2.67% (Ø12-5%) and decreases of 3.18% (Ø12-10) and 4.4% (Ø12-15%) were obtained. When the yield stiffness values were analyzed, the highest stiffness value was observed in the Ø12-0% specimen (9.73 kN/mm), while the lowest stiffness value was observed in the Ø12-15% specimen (7.3 kN/mm). That is, the yield stiffness value decreased by 24.9% compared to the reference specimen with the use of 15% of waste rubber aggregates in concrete.

In terms of energy absorption capacity and ductility, the amount of area under the load–displacement graphs is important. The ductility value generally increased in proportion to the improvement in the rubber proportion. In light of this, the addition of 5% rubber resulted in the behavior that was most similar to that in the reference beam.

The maximum load level was obtained as 127.73 kN at a displacement value of 11.15 mm in the experiment where tensile reinforcement was selected as Ø10, as observed in Figure 5b. This was the case in the case of a reference beam that had 0% waste tires. Both the heaviest load that could be carried and the amount of displacement that corresponded to this load were found to decrease simultaneously as the tire proportion increased. There is a fairly strong relationship between the values obtained at a 5% tire ratio and the findings acquired from the reference beam. It was established that the highest load that could be carried and the displacement measurements that corresponded to this capacity were 11.11 mm and 125.11 kN (Ø10-5%), 9.06 mm and 103.90 kN (Ø10-10%), and 8.11 mm and 95.28 kN (Ø10-15%) for 5%, 10%, and 15% tire proportions, respectively. The stiffness values corresponding to the maximum load value were determined as 11.45 kN/mm (Ø10-0%), 11.25 kN/mm (Ø10-5%), 11.46 kN/mm (Ø10-10%), and 11.75 kN/mm (Ø10-10%). Compared to the reference sample (Ø10-0%), the Ø10-15% and Ø10-10% samples increased by 2.57% and 0.03%, respectively, while Ø10-5% decreased by 1.74%. When the yield stiffness value was analyzed, increases of 20.2% (Ø10-5%), 7.8% (Ø10-10%), and 5.1% (Ø10-0%) were realized compared to the reference sample. In general, it was observed that with the increase in waste rubber aggregates in the concrete, the energy consumption capacity decreased. This is related to the fact that the fracture mechanism of the beam tends towards shear fracture rather than bending. The ductility value increased in proportion to the increase in the rubber proportion. By using 5% rubber, the behavior that was most similar to the reference beam was achieved. This is similar to the studies in which the tensile reinforcement was chosen to be Ø12.

**Figure 6:**
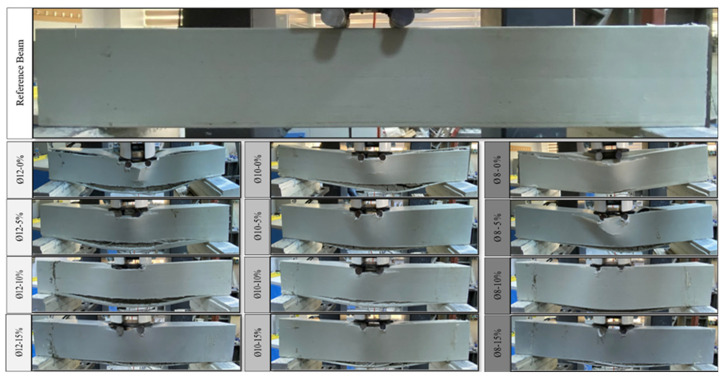
Demonstrating the reference and damage views for the tested specimens.

When the tensile reinforcement was chosen to be Ø8, the highest load level that was achieved was 122.03 kN, and the measurement of displacement was 10.40 mm. This was achieved in the case of a reference beam (Ø8-0%) that did not include any waste tires. It was found that the highest load that could be carried and the amount of displacement that corresponded to this load decreased as the tire proportion increased, except for the Ø8-5% sample. These outcomes are comparable to other studies. There is an extremely strong correlation between the data acquired at a 5% tire proportion and the findings obtained from the reference beam. As per the results of the experiment, the highest load and the displacement values associated with it were ascertained to be 11.51 mm and 116.31 kN (Ø8-5%), 9.92 mm and 104.34 kN (Ø8-10%), and 9.99 mm and 96.39 kN (Ø8-15%) for tire ratios of 5%, 10%, and 15%, respectively. While the stiffness of the reference specimen (Ø8-0%) corresponding to the maximum load value was 11.72 kN/mm, the stiffness values of the specimens containing 5%, 10%, and 15% waste rubber aggregates were determined as 10.09 kN/mm (Ø8-5%), 10.52 kN/mm (Ø8-10%), and 9.64 kN/mm (Ø8-15%), respectively. The reductions in stiffness compared to the reference specimen (Ø8-0%) were found to be 13.88% (Ø8-5%), 10.32% (Ø8-10%), and 17.76% (Ø8-15%). With an increase in the rubber proportion, there was a corresponding gain in ductility. For the 5% waste rubber aggregate content, the energy consumption capacity corresponding to the maximum load, the plastic energy consumption capacity, and the total energy consumption capacity of the specimen increased, while a decrease in yield energy consumption capacity was observed. For 10% and 15% waste rubber aggregate contents, a gradual decrease was observed in all energy consumption capacities. As a result, adding 5% rubber to the tensile reinforcement produced the behavior that was closest to the reference beam. The findings that were achieved in the investigation using a 5% waste rubber aggregate proportion with the reference beam are highly similar to those chosen for Ø12 and Ø10. Figure 6 presents a damage view of all beam specimens after bending tests during the investigation.

### 3.3. Variations in the Effect of the Tension Reinforcement Proportion on Waste Rubber Aggregates

**Figure 7:**
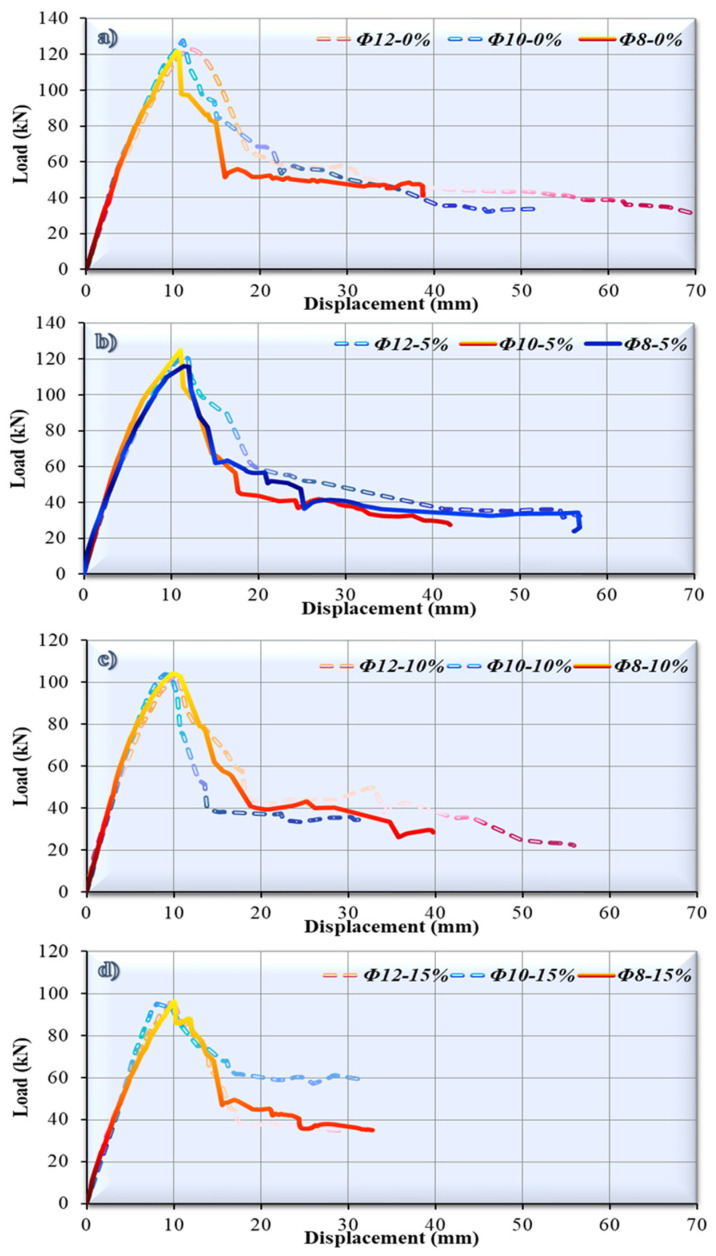
Graphs demonstrating the relationship between load and displacement for the tested specimens.

This section provides a detailed assessment of the impact of varying levels of tension reinforcing on the bending characteristics of RCBs. Experimental findings indicate the presence of significant bending cracks in the reference RCB due to the vertical load being applied. Figure 7 demonstrates that when RCBs are subjected to heavy loads, significant shear cracks may be observed, as well as exceptional bending cracks. It is possible to obtain a load–displacement graph for RCBs, as shown in Figure 7. Based on Figure 7, bending gradually rose in a straight line until it reached a specific point. The end of this straight line corresponds to 123 kN, 127.73 kN, and 122.03 kN for Ø12-0%, Ø10-0%, and Ø8-0%, respectively. Subsequently, bending values of 12 mm, 11.15 mm, and 10.40 mm can be observed at the maximum vertical load for the Ø12-0%, Ø10-0%, and Ø8-0% specimens, respectively. The greatest bending values observed at the final stage of the experiment were 71.64 mm, 51.55 mm, and 38.73 mm for Ø12-0%, Ø10-0%, and Ø8-0%, respectively. At these bending values, the RCBs have already reached their load-carrying capacity. The Ø10-0% specimen stands out with its maximum load-carrying capacity. However, the Ø8-0% specimen stands out with the maximum stiffness value (11.73 kN/mm) corresponding to the maximum load.

Additionally, 122.22 kN, 125.11 kN, and 116.31 kN were recorded for Ø12-5%, Ø10-5%, and Ø8-5%, respectively, as the ratio of waste rubber rose from 0% to 5%. At the maximum vertical load, the Ø12-5% specimen exhibits a bending value of 11.6 mm, the Ø10-5% specimen exhibits a bending value of 11.11 mm, and the Ø8-5% specimen exhibits a bending value of 11.51 mm. During the final phase of the experiment, the highest bending values that were recorded were precisely 56.88 mm, 41.98 mm, and 56.94 mm for Ø12-5%, Ø10-5%, and Ø8-5%, respectively. In general, the Ø10-5% specimen has both the highest load-carrying capacity and the lowest displacement value corresponding to the maximum load. This may suggest that the Ø10-5% specimen performs better in terms of both strength and yield stiffness.

For the 10% waste rubber ratio, the bending results were recorded as 104.24 kN, 103.90 kN, and 104.34 kN for 12–10%, 10–10%, and 8–10%, respectively. These values were acquired by bending the beam samples at the conditions that were given. Under the highest vertical load, the Ø12-10% specimen shows a deflection of 10.5 mm, the Ø10-10% specimen shows a deflection of 9.07 mm, and the Ø8-10% specimen shows a deflection of 9.92 mm. In the last stage of the experiment, the most significant measurements/maximum displacements for bending were exactly 55.97 mm, 31.51 mm, and 39.81 mm for 12-5%, 10-5%, and 8-5%, respectively. The Ø10-10% specimen has the lowest value in both the maximum load level and the stiffness value corresponding to the maximum load value. However, the Ø8-10% specimen stands out as having the maximum load-carrying capacity and stiffness.

**Figure 8:**
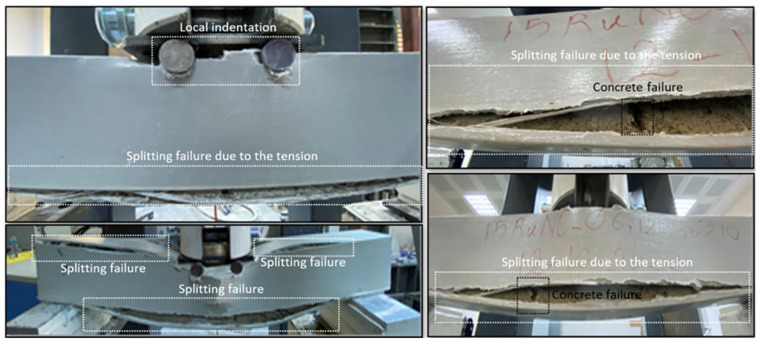
Damage observed in the composite beam.

As the ratio of waste rubber rose from 10% to 15%, it was found that 96.72 kN, 95.28 kN, and 96.39 kN were recorded for Ø12-15%, Ø10-15%, and Ø8-15%, respectively. The specimens Ø12-15%, Ø10-15%, and Ø8-15% demonstrate bending values of 9.87 mm, 8.11 mm, and 9.99 mm, respectively, at the maximum vertical load. For Ø12-15%, Ø10-15%, and Ø8-15%, the most important bending measurements made during the last phase of the experiment were precisely 30.00 mm, 31.78 mm, and 32.75 mm, respectively. Local damage appearances during experimental bending research are given in Figure 8. The load-carrying capacities of the specimens are approximately similar. However, while the maximum load-carrying capacity was observed in the Ø12-15% specimen, the stiffness value corresponding to the maximum load was highest in the Ø10-15% specimen.

**Figure 9:**
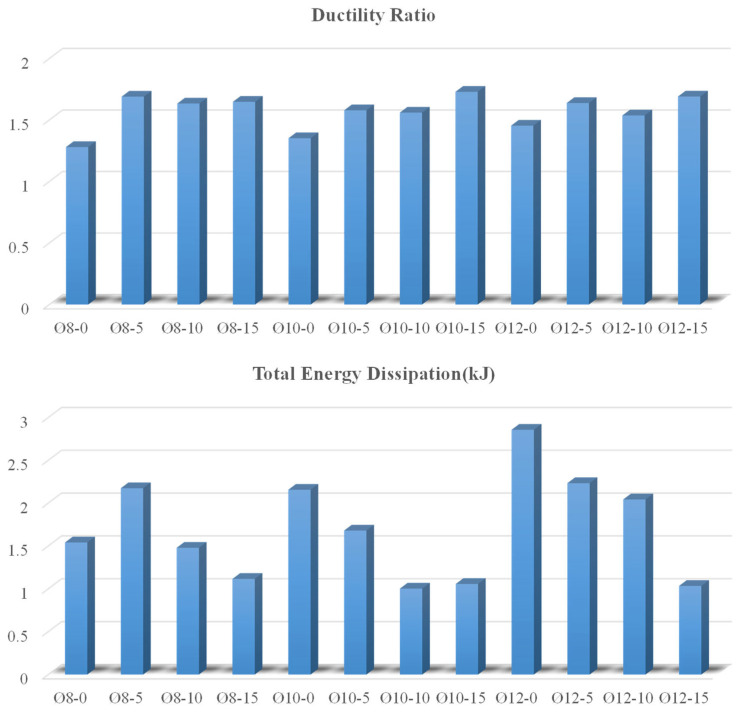
Total energy dissipation and the ductility ratio of the beams.

The general damage observed in the Pultruded GFRP composite beams as a result of the experimental study is shown in Figure 9. As can be seen, the material with the pultruded GFRP profile delayed but did not prevent the occurrence of flexural and shear damage observed in the reinforced concrete beam. Generally, the first instance of damage was observed in the flexural region of the beam, and splitting damage occurred in the pultruded material. As the vertical load increased, splitting damage continued to expand, and web-flange separation due to splitting was observed in the compression zone of the beam. Meanwhile, both shear damage and bending damage have already occurred in the reinforced concrete beam, and local indentation failure was also observed at the beam’s loading point.

The test results obtained for each specimen as a result of the vertical loading tests are given in Table 3 and Table 4. The parametric results given in Table 3 and Table 4 were obtained based on the load–displacement curve. Symbolizing the peak point derived from the load–deformation graph, the P_max_ value is defined here. Furthermore, this point’s deformation amount represents the deformation amount that was achieved in response to the P_max_ value. The point P_u_, which corresponds to the portion of the curve where the load first reaches the value of 0.85 P_max_, is represented in the ascending portion of the curve until the peak. Stiffness is defined as the slope of the linear portion at the outset of the load–displacement curve. A material’s capacity to endure substantial plastic deformation prior to rupture or failure is also defined as ductility. In general, the term “higher ductility” refers to a structural element’s capacity to endure substantial deformations prior to fracture. Equation- is defined as ductility. In this context, the ultimate deformation is denoted by Δ*u*, while the yield deformation is known as Δ*y*.

(1) μ=∆u∆y

On this curve, the maximum load value (P_max_), the load value corresponding to 85% of the maximum load (0.85 P_max_), the yield (δ_y_), the ultimate displacement value (δ_u_) for ductility, and the ductility ratio (δ_u_/δ_y_) are calculated and given in Table 3 Also, the maximum displacement value and the energy dissipation values (0.85 P_max_, P_max_, plastic, and total) for each specimen are calculated and given in Table 4. Finally, the ductile behavior of each specimen is indicated in Table 4.

As illustrated in Figure 9, calculated beam samples’ calculated ductility ratios and total energy dissipation values are provided. In a reinforced concrete beam, the failure energy, ductility, and fracture energy can be initially enhanced by the residual rubber content. Figure 9 provides a great representation of this pattern. Nevertheless, the ultimate compression/tensile/axial strength and rigidity values are observed to decrease as the residual rubber content within the reinforced concrete beam increases. Because of the weaker ITZ between the rubber and the concrete, water absorption in the mixture rises as the amount of rubber in the concrete increases. Although rubber’s hydrophobic properties help resist water to a certain degree, adding it to concrete results in more voids and a less thick matrix. The formation of a compact cement material is disrupted by rubber, which functions as a spacer, resulting in increased permeability and water absorption. Adhesion issues may arise as a result of reduced strength, increased porosity of the rubber core, agglomeration of coated particles, compatibility with the cement material, or insufficient coating thickness, which can slightly increase water absorption [26]. As a result of this behavior, as can be shown in Figure 9, the total absorbed energy, or the area under the load–displacement curve, first rises but subsequently tends to decrease. This is because of the behavior described above. In other words, an optimal range of values for the quantity of waste rubber used is determined. The operation of this mechanism is as follows: The waste rubber particles disperse crack propagation. That is, they allow the microstructure to be displaced or plasticized. Consequently, this results in a slower occurrence of fractures while simultaneously enhancing displacement capacity. Specifically, this growth has a beneficial impact on energy capacity. Because the elastic modulus of waste rubber particles is much lower than the modulus of the concrete aggregate [27], the drop in strength and stiffness may be attributed to this significant difference. Using a significant quantity of rubber causes the connective tissue in concrete to become more fragile. The maximum load is thus decreased as a result of this. Table 3 presents the load decrease in a straightforward and concise manner.

## 4. Conclusions

The flexural behavior of reinforced concrete beams with the addition of waste rubber fiber aggregates was investigated in a series of experimental studies. Concrete mixtures with different proportions (5%, 10%, and 15%) of waste rubber fiber aggregates were prepared, and the behavior of composite beams formed with pultruded GFRP profiles was investigated. Four-point bending tests were carried out using reinforcements of different diameters (Ø8, Ø10, and Ø12). The specific results obtained as a result of this experimental study are given below.

- Firstly, cube, cylinder, and flexural tests were carried out in this study. With the use of the 15% waste rubber aggregate, cube compressive strength, cylinder tensile strength, and flexural strength decreased by 27.5%, 50%, and 47.6%, respectively, compared to the reference specimens.
- As a result of the four-point bending tests on the beams, the inclusion of waste rubber aggregates in the concrete mixtures at the rates of 10% and 15% affected the mechanical properties of the concrete and significantly reduced the maximum load-carrying capacity. However, it was determined that there was an improvement in terms of ductility compared to the reference beams.
- It is understood that the best balance between strength and ductility is achieved, and the performance closest to the reference beams is realized when the waste rubber aggregate ratio is 5%. It can be said that the Ø10-5% specimen shows better performance in terms of both strength and yield stiffness.
- As a result of the tests, it was determined that the beams with the Ø10 reinforcement had both higher load-carrying capacity and higher yield stiffness when using concrete with 5% waste rubber aggregate.
- When the ratio of the waste rubber aggregate is 15%, although the maximum load-carrying capacity decreases with increasing longitudinal reinforcement ratios (Ø12-15% > Ø10-15% > Ø8-15%), the approximate load-carrying capacities of the specimens are similar (Ø12-15% = 96.72 kN > Ø10-15% = 95.28 kN > Ø8-15% = 96.39 kN).
- It was observed that the overall energy consumption capacities for all tested beams generally decreased with the increase in the proportion of waste rubber aggregates. This can be explained by the fact that shear damage in the beam occurs more quickly, and the area under the load–displacement curve decreases with the increase in the amount of waste rubber aggregates.
- It was evaluated within the scope of this experimental study that waste rubber aggregates to be used in reinforced concrete beams can provide environmental and economic benefits at certain ratios (especially at the 5% level).
- Common failures observed in pultruded GFRPs were splitting at the beam’s loading point, web-flange separation, and local notch failure.
- The 5% hybrid ratio is shown to produce the most advantageous overall response, according to a quantitative synthesis of many measures, including strength, ductility, and energy. Ratios exceeding 5% alter the response profile, inducing increased stiffness, reduced deformability, and earlier onset of localization. Consequently, in line with the planned hybrid design goal, the 5% arrangement offers the best balance between strength gain and deformation capacity.

As a result of this study, it is understood that although waste rubber aggregates provide environmental benefits in concrete mixtures, their use should be limited in applications with high strength requirements. For future studies, it can be said that a 5% waste rubber aggregate ratio is the most appropriate balance between strength and ductility enhancement. Although the use of rubber aggregates resulted in a general increase in ductility, albeit an insufficient one, the bending behavior of the beams was controlled by an abrupt shear failure mechanism, particularly at higher rubber contents (10% and 15%). This brittle fracture mode, which limits the ultimate load and displacement capacity, indicates that the potential ductility advantages of rubber aggregates cannot be fully exploited due to insufficient shear resistance. The 5% rubber content provided the best balance, demonstrating both the highest load-carrying capacity among the rubber-modified samples and the most significant improvement in deformation behavior before early shear failure. These results provide an important contribution to the development of sustainable construction materials by integrating waste rubber aggregates into concrete mixtures. Finally, future research efforts are required to fully realize the ductility potential inherent in rubber-reinforced hybrid beams. In particular, detailed analytical investigations focusing on the shear capacity of rubber-reinforced concrete under the confinement effect provided by the GFRP pultrusion profile are essential. Developing validated analytical design equations for the shear strength of these unique hybrid sections will be critical to ensure that the structural system’s response is governed by ductile flexural failure rather than the brittle shear mechanisms observed in the current study. This will ultimately enable designers to safely utilize the increased deformation capacity offered by waste rubber aggregates.

## Figures and Tables

**Table 1:** Chemical and physical properties of CEM I 42.5 R cement.

Chemical Properties	Obtained Values	TS EN 197-1	
		Most	
Loss on Ignition (%)	2.76	5.00	
Insoluble Residue (%)	0.35	5.00	
Sulfur trioxide (SO_3_) (%)	3.4	4.00	
Chloride (Cl) (%)	0.028	0.10	
K_2_O (%)	0.64		
Na_2_O (%)	0.34		
**Physical Properties**	**Obtained values**	**TS EN 197-1**	
		**Least**	**Most**
2-Day Compressive Strength (MPa)	27.2	20	
28-Day Compressive Strength (MPa)	49.7	42.5	62.5
Initial Setting Time (min)	135	60	
Volume Expansion (mm)	1		10

**Table 2:** Cube strengths, cylinder-splitting strengths, and beam bending strengths.

Mix	Slump (cm)	Splitting Tensile Strength (MPa)	Average Splitting Tensile Strength (MPa)	Bending Load (kN)	Bending Strength (MPa)	Average Bending Strength (MPa)	Compressive Strength (MPa)	Compressive Strength Average (MPa)
RuNC	19	2.24	2.31	14.06	8.44	8.270	22.442	22.86
		2.51		13.84	8.30		23.288	
		2.20		13.46	8.07		22.836	
5RuNC	15	1.41	1.58	7.85	4.71	5.257	12.751	12.65
		1.55		9.16	5.49		12.250	
		1.79		9.28	5.57		12.938	
10RuNC	14	1.23	1.24	6.42	3.85	6.013	8.179	8.42
		1.23		12.35	7.41		8.258	
		1.27		11.30	6.78		8.824	
15RuNC	11	1.06	1.18	10.09	6.05	4.330	6.100	6.32
		1.22		6.02	3.61		6.337	
		1.27		5.55	3.33		6.516	

**Table 3:** Test results of composite beams.

Test Specimens	P_max_ (kN)	Displacement at P_max_ (mm)	Stiffness at (P_max_) (kN/mm)	Pu (0.85 P_max_) (kN)	Displacement at Yield δ_y_ (mm)	Stiffness at Yield (0.85 P_max_) (kN/mm)	δ_u_ (mm)	Ductility Ratio
Ø8-0	122.035	10.400	11.729	103.730	8.562	12.115	10.958	1.280
Ø8-5	116.314	11.517	10.099	98.867	7.878	12.550	13.310	1.690
Ø8-10	104.348	9.92	10.519	88.696	6.603	13.433	10.790	1.634
Ø8-15	96.392	9.994	9.646	81.934	7.322	11.191	12.067	1.648
Ø10-0	127.729	11.149	11.457	108.570	8.892	12.210	12.024	1.352
Ø10-5	125.108	11.114	11.257	106.342	7.244	14.680	11.436	1.579
Ø10-10	103.903	9.0664	11.460	88.318	6.708	13.166	10.467	1.560
Ø10-15	95.280	8.108	11.751	80.988	6.311	12.833	10.910	1.729
Ø12-0	123.000	12.000	10.250	104.550	9.730	10.745	14.150	1.454
Ø12-5	122.122	11.604	10.524	103.804	8.230	12.613	13.483	1.638
Ø12-10	104.244	10.504	9.924	88.607	7.463	11.873	11.471	1,537
Ø12-15	96.721	9.877	9.792	82.213	7.300	11.262	12.342	1.691

**Table 4:** Energy dissipation capacity of composites beams.

Test Specimens	Maximum Displacement (mm)	Energy Dissipation at P_max_ (kJ)	Energy Dissipation at 0.85 P_max_ (kJ)	Plastic Energy Dissipation (kJ)	Total Energy Dissipation (kJ)	Ductility Level Sufficient/ Deficient
Ø8-0	38.738	0.720	0.509	1.032	1.541	Deficient
Ø8-5	56.942	0.841	0.444	1.730	2.174	Deficient
Ø8-10	39.819	0.639	0.318	1.160	1.479	Deficient
Ø8-15	32.752	0.557	0.322	0.794	1.116	Deficient
Ø10-0	51.552	0.825	0.553	1.604	2.156	Deficient
Ø10-5	41.980	0.844	0.407	1.273	1.680	Deficient
Ø10-10	31.511	0.050	0.318	0.684	1.003	Deficient
Ø10-15	31.787	0.406	0.247	0.810	1.057	Deficient
Ø12-0	71.638	0.840	0.518	2.338	2.857	Deficient
Ø12-5	56.880	0.853	0.470	1.763	2.233	Deficient
Ø12-10	55.974	0.669	0.374	1.669	2.043	Deficient
Ø12-15	30.000	0.561	0.331	0.703	1.034	Deficient

## Data Availability

The original contributions presented in the study are included in the article. Further inquiries can be directed to the corresponding author.
